# Implementation of a non-separation pathway for late preterm infants in a tertiary care center: a prospective feasibility study

**DOI:** 10.3389/fped.2026.1919628

**Published:** 2026-09-22

**Authors:** Elien Janssens, An Eerdekens

**Affiliations:** 1 Department of Pediatrics and Neonatology, Residency Program, University Hospital Leuven, Leuven, Belgium; 2 Neonatology Department, University Hospital Leuven -KU Leuven, Leuven, Belgium

**Keywords:** breastfeeding, family-centered care, late preterm infant, neonatal outcomes, non-separation, rooming-in, skin-to-skin contact

## Abstract

**Background:**

Late preterm infants (34 + 0–36 + 6 weeks' gestation) are at increased risk of feeding difficulties, hypoglycemia, hypothermia, and early mother-infant separation. Although skin-to-skin contact and rooming-in are recommended components of family-centered neonatal care, evidence regarding the implementation of integrated non-separation strategies in late preterm infants remains limited. This study evaluated the implementation, feasibility, safety, and early clinical outcomes of a Non-Separation Pathway in a tertiary care setting.

**Methods:**

We conducted a prospective observational cohort study after implementation of a Non-Separation Pathway at a Belgian tertiary university hospital. Mother-infant dyads with infants born between 34 + 0 and 36 + 6 weeks' gestation were enrolled between April 2023 and March 2024. The pathway consists of three interconnected components: (1) immediate and sustained delivery-room SSC, (2) continuous rooming-in, and (3) structured feeding support according to a standardized protocol based on Academy of Breastfeeding Medicine. Primary outcomes were pathway implementation, feasibility, and safety, with complete non-separation as the primary implementation outcome. Secondary outcomes included feeding practices, hypoglycemia, weight loss, length of stay, and feeding status at discharge.

**Results:**

Sixty-nine mother-infant dyads were included. Mean gestational age was 35.6 weeks and mean birth weight was 2,502 g. Complete non-separation was achieved in 37.3% of dyads, delivery room skin-to-skin contact in 82.6%. Vaginal birth (aOR 5.12, 95% CI 1.51–17.39) and higher gestational age (aOR 2.14, 95% CI 1.06–4.35) were independently associated with complete non-separation. Respiratory support was independently associated with lower odds of delivery-room skin-to-skin contact (aOR 0.04, 95% CI 0.004–0.41). Hypothermia occurred in 18.8% of infants and was associated with surrogate skin-to-skin contact (aOR 6.67, 95% CI 1.71–26.03). At discharge, 75.4% of infants received any mother's milk and 34.8% were exclusively breastfed.

**Conclusions:**

Implementation of the Non-Separation Pathway demonstrated that delivery-room SSC was feasible for most late preterm infants in a tertiary neonatal setting and was not associated with major safety concerns. However, complete non-separation throughout hospitalization was achieved in only 37% of dyads and remained constrained by respiratory support requirements, cesarean delivery, maternal and neonatal complexity, limited intermediate-care capacity, and the absence of rooming-in facilities in the NICU.

## 1 Introduction

Late preterm infants (LPIs), defined as infants born between 34 + 0 and 36 + 6 weeks' gestation, account for the majority of preterm births worldwide. Although often considered clinically stable, they remain physiologically immature and are at increased risk of feeding difficulties, hypoglycemia, hypothermia, jaundice, prolonged hospitalization, and readmission compared with term infants (1, 2).

LPIs are also frequently separated from their parents shortly after birth. Such separation may interfere with parent–infant bonding and early breastfeeding and conflicts with the principles of family-centered neonatal care (3–5). Skin-to-skin contact (SSC), rooming-in, and kangaroo mother care are key strategies for promoting mother-infant proximity. Accordingly, the World Health Organization recommends immediate SSC and the avoidance of unnecessary mother-infant separation whenever clinically feasible (6).

Although the benefits and feasibility of SSC have been demonstrated in several preterm populations (7, 8), maintaining continuous mother-infant proximity in LPIs presents specific implementation and safety challenges. Respiratory transition difficulties, cardiorespiratory instability, impaired thermoregulation, and the need for monitoring or respiratory support may interrupt SSC or necessitate admission to a separate neonatal unit. In addition, limited space, staffing, monitoring capacity, and coordination between obstetric, anesthetic, and neonatal teams may result in separation even when the infant is clinically stable (9). Maternal pain, fatigue, limited mobility, and postoperative recovery, particularly after cesarean delivery, may further restrict early and sustained proximity (10).

Feeding problems are particularly common in LPIs because of immature suck–swallow coordination, reduced feeding endurance, and delayed establishment of effective breastfeeding. As a result, breastfeeding rates are consistently lower among LPIs than among term-born infants (11, 12). Given the established benefits of mother's own milk for neonatal health and neurodevelopment, international guidelines recommend targeted feeding support and breastfeeding promotion for LPIs (1, 13).

Despite increasing support for SSC, rooming-in, and breastfeeding assistance, prospective studies evaluating their implementation as a continuous non-separation pathway from birth throughout hospitalization remain limited in LPIs, particularly in tertiary care settings. Therefore, we conducted a prospective cohort study to evaluate the implementation, feasibility, and safety of a Non-Separation Pathway. This comprehensive multidisciplinary model aimed to maintain mother-infant proximity throughout the care trajectory by integrating immediate and continuous SSC, 24-hour rooming-in, and structured feeding support.

## 2 Methods

### 2.1 Study design and setting

This prospective observational cohort study evaluated the implementation of a Non-Separation Pathway for late preterm infants at University Hospitals Leuven (UZ Leuven), Belgium. The protocol was introduced as standard clinical practice in April 2023 and was applied to all eligible mother-infant dyads. Data were collected prospectively between April 2023 and March 2024. The study was conducted in a tertiary perinatal center comprising a 36-bed neonatal intensive care unit (NICU), a neonatal intermediate care unit with rooming-in facilities, and a maternity ward. University Hospitals Leuven is a university-affiliated tertiary referral center providing specialized obstetric and neonatal care for high-risk pregnancies and newborns. The center receives referrals for pregnancies complicated by maternal medical conditions, threatened preterm birth, multiple gestations, fetal growth restriction, and suspected or confirmed congenital anomalies. Consequently, the patient population is characterized by a higher prevalence of maternal and neonatal complexity than would typically be encountered in secondary-care or community maternity settings. Written informed consent was obtained from parents before delivery or during the first postnatal week.

### 2.2 Participants

Mother-infant dyads were eligible for inclusion when the infant was born between 34 + 0 and 36 + 6 weeks' gestation during the study period. Exclusion criteria were major congenital anomalies, maternal substance abuse, triplet pregnancies, and postnatal transfer from another hospital. Otherwise eligible dyads were not enrolled when written informed consent could not be obtained within the predefined enrollment period. This was primarily attributable to logistical constraints, including insufficient time between identification of eligibility and delivery and competing demands within obstetric care, and language barriers, rather than parental refusal to participate.

### 2.3 The non-separation pathway

The intervention was a comprehensive, multidisciplinary Non-Separation Pathway designed to maintain mother-infant proximity from birth throughout hospitalization. The pathway consists of three interconnected components: (1) immediate and sustained delivery-room SSC, (2) continuous rooming-in whenever medically possible, and (3) structured feeding support.

SSC could be postponed or interrupted when the clinical condition of the mother or infant precluded its safe continuation. Neonatal indications included the need for resuscitation, cardiorespiratory or hemodynamic instability, or another urgent diagnostic or therapeutic intervention that could not safely be provided during SSC. Maternal indications included clinical instability, postoperative complications, or an inability to participate safely in SSC. Decisions to postpone or interrupt SSC were made by the responsible obstetric and neonatal care teams.

NICU admission, and consequently mother-infant separation, was considered medically indicated in the presence of clinical conditions requiring intensive monitoring or treatment, including significant respiratory distress requiring advanced respiratory support, hemodynamic instability, severe hypoglycemia, suspected sepsis requiring intensive evaluation, or other conditions necessitating NICU-level care. Clinically stable infants could also be admitted to the NICU when no bed was available in the intermediate-care unit. These admissions were classified as capacity-related organizational separations rather than medically indicated separations.

Structured feeding support was provided according to a standardized feeding protocol based on recommendations from the Academy of Breastfeeding Medicine.

Before implementation, multidisciplinary education and training sessions were provided to physicians, midwives, and nurses working in the delivery room, maternity ward, neonatal intermediate-care unit, and NICU. Training covered the rationale and components of the pathway, eligibility and safety criteria, practical organization of delivery-room SSC, neonatal monitoring, escalation of care, and structured feeding support. Detailed pathway and feeding algorithms are presented in Supplementary Figures S1, 2.

During delivery-room SSC, all infants were directly observed by a midwife, and axillary temperature was measured at 15-minute intervals. Infants designated for subsequent admission to the neonatal intermediate-care unit or NICU were additionally monitored by continuous pulse oximetry connected to a central monitoring system.

Complete non-separation, the primary implementation outcome, was defined as immediate maternal SSC after birth, uninterrupted continuation of SSC for at least 60 min, and subsequent rooming-in throughout hospitalization. Delivery-room SSC (DR-SSC) was defined as any SSC initiated during the immediate postnatal stabilization period. Continuous SSC was defined as uninterrupted SSC maintained for at least 60 min. Surrogate SSC referred to SSC provided by another parent or caregiver when maternal SSC was temporarily not possible.

### 2.4 Data collection

Maternal, obstetric, and neonatal characteristics were prospectively recorded during hospitalization. Maternal variables included age, parity, delivery mode, antenatal steroid exposure, and selected medical history. Neonatal variables included gestational age, birth weight, Apgar scores, respiratory support requirements, admission location, complications during hospitalization, feeding characteristics, and discharge outcomes.

Achievement of each pathway component, rooming-in status, admission location, and reasons for NICU admission were obtained from the electronic health record. The initiation and duration of SSC were documented by healthcare professionals and independently reported by mothers using a brief questionnaire.

### 2.5 Outcomes

The primary outcomes were implementation, feasibility, and safety of the Non-Separation Pathway. Complete non-separation was the primary implementation outcome.

Additional implementation and feasibility outcomes included:

- achievement of delivery-room SSC
- achievement of immediate SSC
- achievement of continuous SSC lasting at least 60 min
- rooming-in during the hospital stay
- medically indicated and organizational reasons for separation

Safety outcomes included:

- adverse events occurring during SSC
- hypothermia, defined as an admission temperature <36.5 °C
- clinical instability requiring interruption of SSC
- Apgar scores at 1, 5, and 10 min

Secondary outcomes included feeding practices, use of supplemental feeding, hypoglycemia, weight loss, length of hospital stay, postmenstrual age at discharge, discharge weight, and feeding status at discharge.

### 2.6 Statistical analysis

As this study evaluated the implementation of a newly introduced clinical care pathway, no formal sample size calculation was performed. Statistical analyses were considered exploratory. Continuous variables were assessed descriptively and are presented as mean [standard deviation (SD)] or median [interquartile range (IQR)] as appropriate. Categorical variables are presented as frequencies and percentages. Group comparisons were performed using Pearson's chi-square test or Fisher's exact test for categorical variables and the Mann–Whitney U test for non-normally distributed continuous variables.

Univariable analyses were performed to identify maternal, neonatal, and organizational factors associated with key implementation outcomes, including delivery-room SSC, complete non-separation, hypothermia, and exclusive breastfeeding at discharge. Variables considered clinically relevant and variables showing evidence of association in univariable analyses were subsequently entered into multivariable logistic regression models. Results are reported as odds ratios (ORs) and adjusted odds ratios (aORs) with corresponding 95% confidence intervals (CIs). A two-sided *p*-value <0.05 was considered statistically significant.

As a sensitivity analysis, all singleton infants and the first-born infant from each twin pregnancy were retained, resulting in a sensitivity cohort of 57 infants from 57 independent pregnancies. The original logistic regression models were then repeated using the same outcomes and predictor variables as in the primary analyses.

Given the limited sample size and the relatively small number of outcome events, multivariable analyses should be regarded as exploratory and hypothesis-generating rather than confirmatory.

### 2.7 Ethics and reporting

The study was approved by the local Research Ethics Committee of University Hospitals Leuven (S 66865). Written informed consent was obtained from the parents of all participating infants before inclusion. The study was conducted in accordance with the principles of the Declaration of Helsinki.

This study is reported according to the Strengthening the Reporting of Observational Studies in Epidemiology (STROBE) statement.

## 3 Results

### 3.1 Study population

Between April 2023 and March 2024, 69 mother-infant dyads were enrolled following implementation of the Non-Separation Pathway (Figure 1). Mean gestational age was 35.6 ± 0.9 weeks and mean birth weight was 2,502 ± 372 g. Twin pregnancies accounted for 34.8% of the cohort, while 62.3% of deliveries occurred by cesarean section. Respiratory support was required in 27.5% of infants and 36.2% were initially admitted to the NICU. Maternal and neonatal baseline characteristics are presented in Table 1.

**Figure 1:**
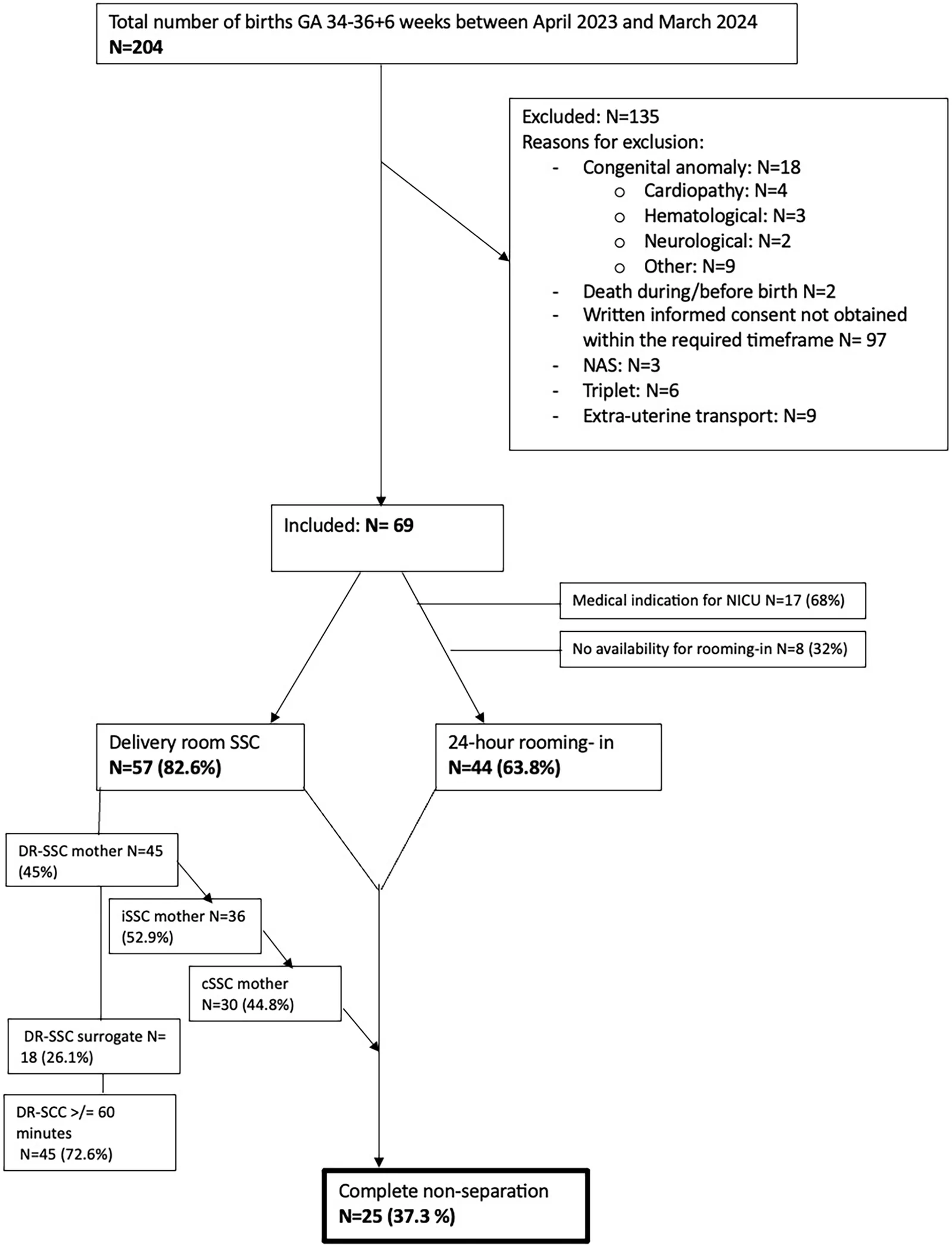
Study flowchart and implementation of non-separation. Participant flowchart showing study inclusion, exclusions, implementation of skin-to-skin care and rooming-in, and achievement of complete non-separation among late preterm infants. SSC, skin-to-skin contact; DR-SSC, delivery room skin-to-skin contact; iSSC, immediate skin-to-skin contact; cSSC, continuous skin-to-skin contact; NICU, neonatal intensive care unit.

**Table 1:** Baseline maternal and neonatal characteristics.

Characteristic	Value
Maternal characteristics	
Maternal age, years	31.4 ± 5.8
Primiparity	34 (49.3)
Cesarean delivery	43 (62.3)
Vaginal delivery	26 (37.7)
Antenatal corticosteroids	18 (26.1)
Indicated preterm birth	43 (62.3)
Neonatal characteristics	
Gestational age, weeks	35.6 ± 0.9
Birth weight, g	2,502 ± 372
Male sex	31 (44.9)
Twin birth	24 (34.8)
Respiratory support	19 (27.5)
Primary NICU admission	25 (36.2)
Hypothermia (<36.5 °C)	13 (18.8)
Hypoglycemia	25 (36.2)
Severe hypoglycemia	17 (23.9)
Weight loss >10%	8 (11.6)

Data presented as mean ± SD or *n* (%).

### 3.2 Implementation and feasibility of the non-separation pathway

Complete non-separation was achieved in 37.3% of dyads. Delivery-room skin-to-skin contact (DR-SSC) was achieved in 82.6% of infants. Immediate SSC with the mother occurred in 52.9% of dyads and continuous SSC for at least 60 min was achieved in 44.8%. Rooming in during the hospital stay was accomplished in 63.8% of dyads, either through the intermediate care unit (37.7%) or on the maternity ward (26.1%) (Table 2).

**Table 2:** Implementation and feasibility outcomes.

Outcome	n/N(%)
Complete non-separation	25/67 (37.3)
Delivery-room SSC	57/69 (82.6)
Immediate maternal SSC	36/68 (52.9)
Continuous SSC ≥60 min	30/67 (44.8)
Surrogate SSC	18/69 (26.1)
Rooming-in achieved	44/69 (63.8)
└ Intermediate care unit	26/69 (37.7)
└ Maternity ward	18/69 (26.1)

Among the 25 infants initially admitted to the NICU, admission was medically indicated in 17 (68%), predominantly because of the need for respiratory support. Eight clinically stable infants (32%) were admitted because no bed was available in the neonatal intermediate-care unit. In 64% of NICU admissions, transfer with the possibility for rooming-in was arranged once the infant was stabilized, no longer required respiratory support, or a bed became available.

Vaginal delivery and higher gestational age were independently associated with complete non-separation. Respiratory support was the principal determinant of unsuccessful DR-SSC. In multivariable analysis, infants requiring respiratory support had substantially lower odds of achieving DR-SSC (aOR 0.04, 95% CI 0.004–0.41; *p* = 0.006) (Table 3).

**Table 3:** Multivariable predictors of key implementation outcomes.

Outcome	Variable	Adjusted OR (95% CI)	*p*-value
DR-SSC	Respiratory support	0.04 (0.004–0.41)	0.006
Complete non-separation	Vaginal delivery	5.12 (1.51–17.39)	0.009
Complete non-separation	Gestational age (per week)	2.14 (1.06–4.35)	0.035
Hypothermia	Surrogate SSC	6.67 (1.71–26.03)	0.006

In a sensitivity analysis restricted to one infant per pregnancy, the effect estimates remained consistent with the primary analyses (Supplementary Table S1).

### 3.3 Safety outcomes

No major safety concerns were identified during implementation of the protocol. One adverse event was reported, consisting of an episode of apnea during surrogate SSC that required temporary interruption of skin-to-skin care for stabilization. No other clinically significant adverse events during SSC were documented.

Hypothermia (admission temperature <36.5 °C) occurred in 13 infants (18.8%). Surrogate SSC was independently associated with an increased likelihood of hypothermia (aOR 6.67, 95% CI 1.71–26.03; *p* = 0.006), whereas no independent associations were observed for gestational age, birth weight, cesarean section, or DR-SSC itself (Table 3).

### 3.4 Feeding outcomes

The first feeding consisted of mother's own milk in 76.8% of infants. During hospitalization, 87.0% received supplemental feeding in addition to or instead of direct breastfeeding. Formula was the most frequently administered supplement (52.2%), followed by a combination of mother's milk and formula (24.6%). Tube feeding was required in 55.1% of infants and intravenous fluid therapy in 31.9%.

Hypoglycemia occurred in 36.2% of infants, with severe hypoglycemia observed in 23.9%. Excessive weight loss exceeding 10% of birth weight was observed in 11.6% of the cohort. At discharge, 34.8% of infants were exclusively breastfed, 47.8% received exclusive mother's milk, and 75.4% received any mother's milk (Table 4).

**Table 4:** Feeding and discharge outcomes.

Outcome	Value
First feed mother's milk	53 (76.8)
Supplemental feeding	60 (87.0)
Formula supplementation	36 (52.2)
Mother's milk + formula	17 (24.6)
Expressed mother's milk only	4 (5.8)
Donor milk ± formula	3 (4.3)
Tube feeding	38 (55.1)
Hypoglycemia	25 (36.2)
Severe hypoglycemia needing intervention	17 (23.9)
Exclusive breastfeeding at discharge	24 (34.8)
Exclusive mother's milk feeding	33 (47.8)
Any mother's milk at discharge	52 (75.4)
Length of stay, days	13.9 ± 7.0
Postmenstrual age at discharge, weeks	37.5 ± 1.0
Discharge weight, g	2,650 ± 301
Discharged home	62 (89.9)

Data presented as mean ± SD or *n* (%).

Receipt of mother's milk as the first feed was associated with exclusive breastfeeding at discharge (OR 11.50, 95% CI 1.41–93.52; *p* = 0.006), whereas infants requiring supplemental feeding were less likely to be exclusively breastfed (*p* < 0.001). Complete non-separation was not significantly associated with exclusive breastfeeding at discharge.

### 3.5 Discharge outcomes

Mean duration of hospitalization was 13.9 ± 7.0 days. Mean postmenstrual age at discharge was 37.5 ± 1.0 weeks and mean discharge weight was 2,650 ± 301 g. Most infants were discharged home (89.9%), while 10.1% were transferred to another hospital before discharge (Table 4).

## 4 Discussion

In this prospective implementation study, delivery-room SSC was achieved in more than four out of five late preterm infants, whereas complete non-separation throughout hospitalization was achieved in only 37% of mother-infant dyads. This distinction demonstrates that successful initiation of SSC does not necessarily translate into sustained mother-infant proximity across the full care trajectory and highlights the importance of organizational and infrastructural factors in addition to clinical eligibility.

No major safety concerns were identified during pathway implementation. One adverse event occurred during surrogate SSC and consisted of an episode of apnea requiring temporary interruption of SSC and neonatal stabilization. Although the study was not powered to formally assess safety, these findings are reassuring and consistent with previous reports supporting early SSC in clinically stable preterm infants (7, 14). Nevertheless, larger studies remain necessary before definitive conclusions regarding safety can be drawn.

Respiratory support emerged as the most important barrier to successful delivery-room SSC, while cesarean delivery and lower gestational age reduced the likelihood of complete non-separation. These findings are consistent with observations by Larsen et al. (2025), who identified neonatal instability and obstetric factors as major determinants of early SSC implementation, and with the broader literature on family-centered neonatal care (3, 4, 15). In a tertiary-care setting, mother-infant separation may occur more frequently because infants are more likely to require respiratory support, intensive monitoring, specialized treatment, or NICU admission, while mothers may require additional postoperative or medical care. Implementation also requires coordination across the delivery room, maternity ward, neonatal intermediate-care unit, and NICU, creating organizational challenges that may be less prominent in lower-acuity settings.

Importantly, separation was not exclusively related to medical instability. Almost one third of initial NICU admissions occurred because no bed was available in the neonatal intermediate-care unit, resulting in potentially avoidable, capacity-related separation. In addition, the current NICU infrastructure does not permit continuous maternal rooming-in. These findings indicate that implementation depends not only on clinical protocols but also on bed capacity, staffing, monitoring facilities, and infrastructure that allows neonatal care to be delivered without separating the mother-infant dyad. Emerging evidence that respiratory support can be maintained during delivery-room SSC further suggests that some clinical barriers may be reduced through adapted equipment and care models (16).

An unexpected finding was the association between surrogate skin-to-skin contact and hypothermia. This association may also be biologically plausible. Bystrova et al. (2007) demonstrated that maternal breast temperature is responsive during early postpartum SSC and closely related to infant temperature, supporting the concept that the maternal chest acts as a dynamic thermoregulatory environment for the newborn (17). Whether comparable thermal adaptation occurs during SSC with another parent or caregiver remains insufficiently studied. Surrogate SSC may remain a valuable strategy when maternal SSC is temporarily impossible, but our findings support careful temperature monitoring and further investigation of optimal thermal protection during this practice.

Feeding challenges remained common despite implementation of the standardized feeding protocol. Nearly nine out of ten infants required supplemental feeding, more than one third experienced hypoglycemia, and more than half required tube feeding during hospitalization. These findings illustrate the persistent nutritional vulnerability of LPIs and are consistent with previous reports emphasizing feeding immaturity as a major challenge in this population (1, 11).

At discharge, 34.8% of infants were exclusively breastfed, 47.8% received exclusive mother's own milk, and 75.4% received at least some mother's own milk. The exclusive breastfeeding rate was comparable to or slightly higher than that reported in some Italian cohorts (18, 19), but lower than the 52.2% reported for preterm infants in Flanders (20) and the 74% reported among late preterm infants in an Australian multicenter cohort (21). These comparisons should be interpreted cautiously because patient characteristics, feeding definitions, discharge timing, lactation support, and supplementation practices differ between studies.

Several factors may have limited exclusive breastfeeding in our cohort. The tertiary-care population included high proportions of twin births and cesarean deliveries, as well as infants requiring respiratory support or neonatal-unit admission. In addition, supplementation was initiated according to predefined clinical and weight-loss criteria. Although intended to reduce the risks associated with insufficient intake, hypoglycemia, and excessive weight loss, early supplementation may have reduced opportunities to establish exclusive breastfeeding.

Complete non-separation was not significantly associated with exclusive breastfeeding at discharge. This finding should not be interpreted as evidence that non-separation has no benefit for breastfeeding. Breastfeeding was a secondary exploratory outcome, and the study was not powered to detect modest differences. Moreover, important determinants of breastfeeding success—including maternal feeding intention, previous breastfeeding experience, socioeconomic circumstances, milk-expression practices, and the intensity of individualized lactation support—were not systematically collected. Previous reviews have demonstrated that successful breastfeeding in LPIs depends on a complex interaction between mother-infant proximity, lactation support, milk expression practices, and clinical stability (12, 22, 23).

This study has several strengths. To our knowledge, few prospective studies have evaluated a comprehensive care pathway that combines non-separation practices with a standardized feeding protocol specifically in LPIs. Furthermore, prospective data collection allowed detailed assessment of implementation outcomes, barriers to non-separation, feeding practices, and early clinical outcomes in a real-world tertiary care setting.

Several limitations should also be acknowledged. First, the study was conducted at a single tertiary center and included a relatively small sample size, limiting statistical power and generalizability. Second, the observational design without a comparison group precludes causal inference regarding the effects of the protocol on breastfeeding, safety, or clinical outcomes. Third, several multivariable analyses were based on a limited number of outcome events and should therefore be considered exploratory.

Fourth, approximately one third of the cohort consisted of twins, reflecting the referral profile and expertise of the center in managing complicated multiple pregnancies. Although twins were analyzed as separate observations in the primary analyses, a sensitivity analysis restricted to one infant per pregnancy yielded comparable estimates, suggesting that within-pregnancy clustering did not materially affect the main findings. Fifth, selection bias cannot be excluded because written informed consent could not always be obtained within the predefined enrollment period, particularly because of logistical constraints or language barriers.

Finally, SSC was interrupted before 60 min in 17% of infants without a clearly documented clinical indication. These interruptions may have reflected workflow constraints, staffing limitations, competing clinical activities, uncertainty regarding responsibilities, or maternal factors such as pain, fatigue, limited mobility, and recovery after cesarean or complicated delivery. However, because the reasons for interruption were not systematically documented, this interpretation remains speculative.

## 5 Conclusion

Implementation of the Non-Separation Pathway demonstrated that delivery-room SSC was feasible for most late preterm infants in a tertiary neonatal setting and was not associated with major safety concerns. However, complete non-separation throughout hospitalization was achieved in only 37% of dyads and remained constrained by respiratory support requirements, cesarean delivery, maternal and neonatal complexity, limited intermediate-care capacity, and the absence of rooming-in facilities in the NICU. Future multicenter comparative studies should prospectively distinguish clinical, organizational, maternal, and family-related reasons for separation, identify modifiable barriers to sustained mother-infant proximity, and determine whether non-separation pathways improve breastfeeding outcomes, neonatal morbidity, parental experience, and longer-term infant outcomes.

## Data Availability

The raw data supporting the conclusions of this article will be made available by the authors, without undue reservation.
