# Supplementary material for: Implementation of a non-separation pathway for late preterm infants in a tertiary care center: a prospective feasibility study
Source: Front Pediatr. 2026 Sep 22;14:1919628. doi: 10.3389/fped.2026.1919628 (PMC13638661; doi:10.3389/fped.2026.1919628)
Supplement: Supplementary file 1 [file Supplementaryfile1.docx]

Supplementary Material

## 1.1 Supplementary Figures

## Supplementary Figure 1. Flowchart outlining clinical decision-making for delivery room skin-to-skin contact, rooming-in, neonatal admission, and transfer procedures according to infant stability, birth weight, gestational age, and maternal condition

**Supplementary Figure 2.** Protocol for initiation and advancement of feeding during hospitalization, including breastfeeding support, monitoring of feeding adequacy, supplementation criteria, and follow-up of weight loss and metabolic complications. Based on ABM. (1)

1.	Boies EG, Vaucher YE. ABM Clinical Protocol #10: Breastfeeding the Late Preterm (34-36 6/7 Weeks of Gestation) and Early Term Infants (37-38 6/7 Weeks of Gestation), Second Revision 2016. Breastfeed Med. 2016;11:494–500.

## 1.2  Supplementary table

| **Outcome** | **Variable** | **Adjusted OR (95% CI)** | **p-value** |
| --- | --- | --- | --- |
| DR-SSC | Respiratory support | 0.019 (0.002–0.178) | <0.001 |
| Complete non-separation | Vaginal delivery | 6.461 (1.785–23.389) | 0.004 |
| Complete non-separation | Gestational age (per week) | 2.181 (1.041–4.572) | 0.039 |
| Hypothermia | Surrogate SSC | 8 (1.85–34.605) | 0.005 |

**Supplementary Table S1:** Sensitivity analysis restricted to one infant per pregnancy, multivariable predictors of key implementation outcomes.
